# Response to: The BRAIN TRIAL: a randomized, placebo controlled trial of a Bradykinin B2 receptor antagonist (Anatibant) in patients with traumatic brain injury - authors' reply

**DOI:** 10.1186/1745-6215-10-111

**Published:** 2009-12-03

**Authors:** Ian Roberts, Haleema Shakur

**Affiliations:** 1 Trials Co-ordinating Centre, London School of Hygiene & Tropical Medicine, Keppel St, London WC1E 7HT, UK

## Abstract

A response to the letter regarding The BRAIN TRIAL: a randomized, placebo controlled trial of a Bradykinin B2 receptor antagonist (Anatibant) in patients with traumatic brain injury, by Mr Vincent Simmon President and CEO of Xytis Inc.

## Letter

We were disappointed to read that [1] Mr Simmon has repeated the claims and allegations about Serious Adverse Events that were withdrawn in full by Xytis during the legal proceedings in the High Court of Justice. We do not think it would be appropriate to consider these claims and allegations here, instead we direct readers to the public documentation surrounding this litigation that are available on the trial website [2].

Mr Simmon correctly points out that the trial manuscript [3] quotes the primary analysis as stated in the protocol rather than the primary objective.

We state clearly in the manuscript that both intention to treat (ITT) and per protocol analyses (PP) were conducted. For the primary end point both analyses are presented but for remainder, only ITT analysis was presented since there was no material difference between them.

Although Xytis sought termination of the trial contract with LSHTM in November 2007, no request was made for the termination of the trial. Indeed, in December 2007, Xytis sought and obtained a High Court injunction preventing the Trial Steering Committee or Data Monitoring Committee from making any recommendations for the termination of the trial.

Mr Simmon admits to having accessed the un-blinded data from the BRAIN trial. This was not permitted according to the trial protocol which states that "None of the persons directly involved in the conduct of the trial will have access to the treatment codes." Neither Ian Roberts nor Haleema Shakur had access to the treatment codes. As members of the Trial Steering Committee Ian Roberts and Haleema Shakur were shown selected tables of un-blinded data by the Data Monitoring Committee in order to judge whether the trial should continue. This was permitted by the trial protocol. However, the allegation that they were un-blinded to the data while queries were on-going and prior to the final data analysis is false.

The HIREOS Scale is a modified version of the Oxford Handicap Scale. Its use in the BRAIN trial was specified in the agreed trial protocol. A paper reporting the predictive validity of the scale has been published [4].

We will not comment on the additional data analyses presented by Mr Simmon since these analyses were not specified in the Statistical Analysis Plan and are not relevant to the trial report.

After a long and expensive legal battle we are pleased that Trials has published the results of the BRAIN trial thus allowing us to meet our ethical obligations to the trial participants that the data are made publicly available.

## Competing interests

The authors declare that they have no competing interests.

## Disclaimer

The factual contents of this paper and the details provided about the clinical trial reported therein have not been verified by BioMed Central Limited ('the publisher') its agents or employees. This article has been published in accordance with this journal's obligation to report all clinical trials, in the public interest. No liability is accepted by the publisher, its agents or employees in connection with the contents of this paper.

## Authors' contributions

This response was written by Ian Roberts and Haleema Shakur.
